# Organ on Chip Technology to Model Cancer Growth and Metastasis

**DOI:** 10.3390/bioengineering9010028

**Published:** 2022-01-11

**Authors:** Giorgia Imparato, Francesco Urciuolo, Paolo Antonio Netti

**Affiliations:** 1 Center for Advanced Biomaterials for HealthCare@CRIB, Istituto Italiano di Tecnologia, Largo Barsanti e Matteucci 53, 80125 Naples, Italy; urciuolo@unina.it (F.U.); Paolo.netti@iit.it (P.A.N.); 2 Department of Chemical, Materials and Industrial Production (DICMAPI), Interdisciplinary Research Centre on Biomaterials (CRIB), University of Naples Federico II, P.leTecchio 80, 80125 Naples, Italy

**Keywords:** cancer on chip, tumor microenvironment, metastasis, organ on chip, 3D tissue

## Abstract

Organ on chip (OOC) has emerged as a major technological breakthrough and distinct model system revolutionizing biomedical research and drug discovery by recapitulating the crucial structural and functional complexity of human organs in vitro. OOC are rapidly emerging as powerful tools for oncology research. Indeed, Cancer on chip (COC) can ideally reproduce certain key aspects of the tumor microenvironment (TME), such as biochemical gradients and niche factors, dynamic cell–cell and cell–matrix interactions, and complex tissue structures composed of tumor and stromal cells. Here, we review the state of the art in COC models with a focus on the microphysiological systems that host multicellular 3D tissue engineering models and can help elucidate the complex biology of TME and cancer growth and progression. Finally, some examples of microengineered tumor models integrated with multi-organ microdevices to study disease progression in different tissues will be presented.

## 1. Introduction

For an extended time, tumor initiation, progression, and metastasis were seen to be simply due to changes within the neoplastic cell population and little attention was given to investigating the biological context, such as TME, in which such neoplastic cells were embedded [1]. The efficiency of genetic approaches, like sequencing, and the use of model organisms to study biological outcomes of mutations, has fostered for several years the concept of cancer as a disease mainly driven by mutations [2]. Recently, the relevance of the TME arose from the observation of the histo-pathological modification found at the interface between putative tumor cells and the surrounding non-neoplastic tissues during carcinogenesis, highlighting that TME is a specialized entity: dynamic, interactive, and constantly changing [1,3]. In addition, it is recognized that cancer cells are able to respond to environmental cues provided by TME [2]. These conclusions sustain a drastic change in the way of conceiving the cancer: from a gene-centric to a dynamic disease featured by complex interactions between cancer cells and their environment [1,3]. This new vision of cancer requires a proportionate change in the models used to study the pathology and develop/test anticancer therapeutics. Classical in vitro cultures of cancer cell lines and tumor spheroids ignore noncancerous parenchymal cells and the tumor stroma, which is composed of extra cellular matrix (ECM), fibroblasts, endothelial, and immune cells, and can make up the bulk (up to 90%) of the total tumor mass [2]. However, the development of optimal surrogate platforms on which to investigate the complex features of cancer cells, such as migration, proliferation, and chemoresistance [4,5], has proven to be very challenging both in vitro and in vivo because of the difficulty in reproducing all the complex tumoral and non-tumoral cell interactions.

Defining optimal in vitro tumor models to mimic the specificity of the TME seems to be of growing interest for the scientific community, demonstrated by the fact that during the last ten years, the number of publications on the topic increased exponentially [1,4]. This is due to the recent progresses in developing many different new techniques that are potentially of great value in the context of 3D tumor models and tumor–TME interaction studies [5,6,7]. Advancements in 3D cell culture systems resulted in the last years in a plethora of 3D cancer in vitro models able to replicate several hallmarks of in vivo disease [8,9,10,11,12,13,14,15]. Recently, the integration of 3D cell/tissue culture systems with microfluidics has conducted to the development of new platforms named microphysiological systems or OOC [16,17,18,19,20,21,22,23]. Today, there exist several microfluidic chips for lung [24,25], liver [26,27], skin [28], gut [29], brain [30]. Such systems, which in the oncology field are often named COC [16,19,21,22,31,32], allow to study the dynamic interplay between cells and their 3D microenvironment capturing several organ level architectures and physiologies such as tissue barriers, mass transport, and vascular perfusion [1]. Moreover, COC systems can integrate patient-derived tumor cells with organ-specific environments that closely approximate the in vivo human tumor environment [2]. Therefore, COC technology represents the most promising tool for cancer studies, providing a unique approach which integrates microfluidics, microfabrication, tissue engineering, and biomaterials research. Moreover, a future integration of COC technology with artificial intelligence (AI) could dramatically revolutionize the oncology research, significantly advancing our understanding of cancer biology, allowing accelerated and cost effective drug discovery [21,33,34,35,36].

Here, we provide an overview of different COC models with a special attention on those are designed to house multicellular 3D models and discuss their use and challenges to investigate the interactions of cancer cells with cellular and non-cellular components of the TME during cancer progression. In addition, we give also an overview of recent progress in the use of multiorgan on chip platform to interconnect different engineered organs and their potentiality in the oncology field [37,38]. Indeed, due to the recent advances in coupling multiple human organ chips to create human body on chip models, the possibility of creating multiorgan models of the metastatic spread of cancer is currently under investigation [16,20,39,40,41,42].

## 2. A Glimpse at Tumor Microenvironment: In Vivo Features and Functions

### 2.1. Cellular Components of the Tumor Microenvironment

It is today widely known that cancer is not only related to cells’ genetic mutations, but it also embroils the complex interactions between the tumor cells and their surrounding tissue [43]. The latter presents specific physical and biochemical features that are involved in regulating neoplastic cell differentiation, proliferation, invasion, and metastasis [43]. Although tumor genetic heterogeneity remains an important barrier to effective cancer elimination, the TME plays an equally pivotal role in cancer initiation, progression and drug resistance, thus representing a promising therapeutic target independent of the plethora of genomic aberrations unique to each tumor [19,32,44]. Therefore, developing an in vitro model to know how cancer cells interact and communicate with their surrounding tissue and the way this interplay controls pathology progression is a vital tool for cancer research.

TME consists of tumor cells, tumor stromal cells including stromal fibroblasts, endothelial cells and immune cells like microglia, macrophages, and lymphocytes and the non-cellular components of the extracellular matrix such as collagen, fibronectin, hyaluronan, laminin, among others [45]. Cancer or tumor cells are those harboring genetic mutations and have the unique attitude of reprogramming their cellular activities to support their rapid proliferation and migration and to counteract metabolic and genotoxic stress during cancer progression (Metabolic features of cancer cells). Normal and cancer cells present differences both in the morphology and in function. Under a microscope, in contrast to normal cells, cancer cells often exhibit much more variability in cell size, have an abnormal shape, both of the cell, and of the nucleus. The latter appears larger and darker since it contains excess DNA. Moreover, while normal cells perform the function they are meant to carry out, cancer cells may not be functional. They do not respond to signals sent from other nearby cells such as the indication to stop growing, and grow even when growth is not necessary, they do not undergo apoptosis and are able to evade the immune system long enough to grow into a tumor. On the other side, the non-malignant cells in the TME have a key role in promoting tumorigenesis in all phases of cancer development and metastasis [45]. The primary non-malignant stromal cells of the TME are cancer-associated fibroblasts (CAFs) and endothelial cells; both these cells actively interact with tumor cells, among themselves, and with the ECM by secreting chemokines, growth factors, enzymes, extracellular vesicles, and miRNAs that regulate the expression of genes and proteins which influence metabolic pathways associated with cancer [46].

CAFs are very abundant within the tumor stroma and are recruited and activated by cancer cells to push ECM remodeling, neo-angiogenesis, proliferation, migration, invasion, metastasis, and affect drug resistance mechanism by releasing growth factors, chemokines, and cytokines. CAFs share many similarities with activated fibroblasts in wound healing; both are usually identified as α-smooth muscle actin (α-SMA)-positive cells. Unlike noncancerous myofibroblasts, CAFs do not revert to their inactivated state and overexpress the platelet-derived growth factor (PDGF) receptor-β, which successively supports their own proliferation [8,47]. Due to their important role in enhancing tumor growth, CAFs are currently taken into account in many studies as primary targets of anticancer therapeutic approaches [48].

Endothelial cells play a fundamental role in tumor angiogenesis, that is, the growth of new vessels from preexisting vascular beds. In healthy tissue, the vasculature is quiescent and endothelial cells are non-proliferative. In TME, pathological unbalanced between pro- and anti-angiogenic factors induce endothelial cells to sprout and initiate angiogenesis in a process called the “angiogenic switch”. Malignant progression of benign tumors is typically associated with an angiogenic switch releases tumors from dormancy and sparks rapid growth of malignant cells in association with new blood vessel formation (https://doi.org/10.1007/s00018-019-03351-7, (accessed on 23 November 2021).

### 2.2. The Extra Cellular Matrix in Tumor Microenvironment

The non-cellular portion of the TME is especially composed of the encompassing ECM that is a complex mixture of structural proteins, glycoproteins, and proteoglycans, which offer not only essential physical scaffolds to take care of tissue structure but also various biochemical signals to modulate cellular functions [49,50,51]. The complex homeostatic equilibrium and dynamical reciprocity existing between the cell and ECM dictates tissue and organ functions. In tumors, the fine balance of the ECM signal is altered inducing the further cancer development and progression. The microenvironmental stimuli, like hypoxia and solid stresses, drive excessive matrix remodeling, leading to a change of the ECM physical properties. The modifications in the amount, composition, or organization of the ECM induce to changes in the properties of the ECM itself, that in turn promotes the formation of a tumorigenic microenvironment [32,52,53]. In such a context, cell growth is promoted by stiffening and cell–cell junction integrity is damaged, impairing lumen formation. Then, non-polarized, disorganized, and invasive colonies deprived by cell–cell junction proteins are formed, representing one of the distinctive feature of cancer [54]. The continuous cross-talking between malignant cells and the TME lead to active ECM remodeling that in turn induces the recruitment of fibroblasts, immune-inflammatory cells, and perivascular cells to encourage neoplastic cell dissemination and invasion to distant organ [32].

Solid tumors contain large extracellular matrix deposits that constitute up to 60% of the tumor mass. Large collagen deposits, together with a high percentage of fibroblast infiltration, result in desmoplasia, which is strongly linked to poor patient prognosis [48].

Other major components of TME are ECM proteins, like collagens, fibronectin, laminin, hyaluronan, tenascin, periostin, elastic fibers, and lots of others. They are highly expressed in metastatic tumor and play important roles in the tumor metastasis niche [49].

### 2.3. The Role of Tumor Microenvironment in Tumor Metastasis

**Figure 1:**
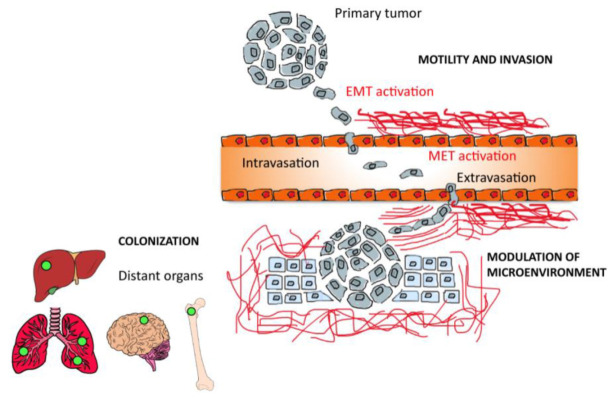
Hallmarks of the metastatic process: Neoplastic cells migrate from the primary tumor and start to invade through surrounding stroma via a multiple motility mechanisms (as single cells via EMT). Single cells once entered into the vasculature (intravasation) roll along the endothelium and selectively adhere to endothelium or stop when the vessel diameter is too small to traverse. Adhesion tumor cells exit vessels (extravasation), the ECM is reorganized and can result in the release of matrikines that affect tumor cell and/or stromal behavior. This activated environment results permissive for proliferation and colonization of secondary sites. Disseminating cells selectively colonize several tissues and the process of further dissemination (i.e., metastasizing from metastases) can occur.

The TME has a relevant role in metastasis, that is, a multifactorial process involving genetic, epigentic, and microenviromental factors in both the primary tumor and the organs that receive the metastatic cells. During the metastatic process, a cancer cell from the primary tumor undergoes the following step: (i) invades locally the surrounding tissue; (ii) modifies its phenotype passing from the epithelial to mesenchymal one by means of the epithelial–mesenchymal transition (EMT) process; (iii) enters in the vasculature of the blood and lymphatic system (intravasation) and then (iv) translocates to the microvascuature of the target tissue (extravasation); (v) invades the target tissue where proliferate and form the secondary tumor (Figure 1). EMT is a key event in promoting tumor cell migration and invasion. It includes a group of biological processes, regulated by a series of transcription factors (i.e., SNAIL, Slug, Twist, and Zeb) that confer on epithelial cells, both normal and neoplastic, properties that are critical for invasion and metastatic spread, increasing their mobility, invasiveness, and the ability to degrade the components of the ECM [55]. Therefore, TME and ECM composition and mechanical properties have a critical role in regulating and promoting EMT process. It has been demonstrated that ECM proteins, including Collagen-I, Fibronectin, and Hyaluronan, are implicated in SNAIL regulation, thus affecting the EMT process. Moreover, ECM remodeling via extracellular Lysyl oxidase, occurring during tumor progression is also implicated in regulating EMT. Simulating in vitro human TME may reveal how EMT occurs and how TME influence the entire metametastatic process. To this aim, advanced in vitro models are necessary to replicate the three-dimensionality, the matrix organization and composition, as well as the cellular heterogeneity of the native TME.

## 3. Tumor Microenvironment in Vitro Modeling

### 3.1. Advanced 3D Systems

In this framework, it is evident, that over simplistic monolayer cultures (2D), largely exploited in the past to accumulate information on cancer cell behavior and still used for the prediction of drug responses, fail in mimicking essential characteristics of tumors and TME and in replicating key patho-physiological events such as the complex process of tumor invasion. Indeed, cells arranged in a monolayer grown in an unnatural context, experiencing artificial cell–substrate interaction unable to mimic the dynamic cell–ECM crosstalk existing in vivo. In addition, cell function (morphology, polarity, and method of division), gene expression, epigenetics, and extracellular receptors presentations of cells in 2D are strongly different compared to the native context [6,19].

In contrast, 3D culture systems have revealed in several studies, their capability of promoting essential biological processes including cell differentiation, proliferation, and morphogenesis establishing clearly their superiority compared to the 2D culture system in the study of complex cellular interactions and as a model for clinical translation [56]. Due to the strong interaction between cancer cells and TME, 3D culture systems raised the more promising model for the study of cancer and the screening of innovative anticancer drugs [40].

ECM composition as well as its properties, such as elasticity, nano-, and microstructure, affect tumorigenesis and need to be considered when designing a 3D in vitro tumor model. Replicating in vitro as close as possible, the ECM composition of organ specific human connective tissues is critical to mimic their structure–function relationship in healthy and diseased conditions. Bissell and colleagues were the first to demonstrate the relevance of the tissue-specific TME in several studies [50,51,57,58]. They demonstrated the different behavior of mammary epithelial cells depending upon the hydrogel in which they were cultured. When grown on laminin-rich reconstituted basement membrane, the cells self-assembled into spherical structures with a central lumen and produced specific protein in response to stimuli, behaving very similarly to normal mammary acini. On the contrary, when the same epithelial cells were cultured in 3D collagen type I gels, the self-assembled spheres failed to form a central lumen and did not produce the specific protein. Interestingly, the researcher’s group demonstrated that the formation of the lumen could be obtained if the mammary luminal epithelial cells were co-cultured with myoepithelial cells that could deposit the basement membrane in situ, suggesting a critical role of the ECM composition in dictating the tissue structure and function [58]. However, recent works [40] demonstrated that exogenous hydrogel such as Collagen, fibrin, Matrigel, while effective in vitro matrices for cells cultures, often fail in recapitulating the diverse biochemical and physical aspects of native tumoral ECM. In this perspective, recently decellularized ECM [59,60], and cell synthesized matrices [12,56,61,62] have been used as 3D matrices for cell culture in order to increase the complexity and increase the pato-physiological relevance of the TME in vitro. Along this line, Hughes et al. extracted and compared ECM from normal human colon tissue and colon tumor metastases and found differences in protein composition and stiffness between the two reconstituted matrices with overrepresentation of several matrix proteins in the tumor ECM as well as an increase in stiffness compared to normal ECM. In an in vitro assay, where tumor cells were co-cultured with endothelial cells and fibroblasts in the reconstituted matrices, vascular network formation and tumor growth were significantly increased in tumor ECM compared to normal ECM demonstrating a severe effect of ECM composition and stiffness on cell behavior within the TME [8]. The production of decellularized matrices, however, presents some limitations both correlated with patient specific variability and with the decellularization process. Indeed, the latter is challenging to ensure tissue intactness after treatment with detergents and enzymes, resulting in low-reproducible method [7]. In the attempt, to more physiologically replicate the natural biochemical environment and the tissue specific architecture, other bioengineering approaches that instruct stromal cells to synthesize and assemble their own ECM (we referred to as endogenous ECM) have been developed [63]. Recently in our work, we demonstrated that in order to produce a 3D tumor stroma model that aspires to mimicking its in vivo counterpart, it is fundamental to replicate the fibroblasts’ ability to elaborate a different ECM depending on their own activation state. Indeed, we arranged normal fibroblasts (NF) and CAF in two different configurations such as spheroid models and engineered microtissues (μTP), and compared the biophysical properties of μTP and spheroids in terms of metabolic activity, mechanical properties, and ECM composition in order to understand which 3D model would better mimic the structure and functions of native tumor stroma microenvironment. We found that the biophysical properties of the stromal spheroid models were insensitive to the nature of the fibroblasts. On the contrary, in the form of stromal microtissues, intrinsic features such as metabolic activity, mechanical properties, and ECM composition, were found to be dependent upon the nature of the fibroblasts, as found in vivo between normal and cancer-activated stroma [64]. In a similar fashion, recently, other sophisticated endogenous ECM based 3D systems combining cancer cells in self-secreted stroma have been developed to emphasize the importance of the organ specific TME and cell–cell crosstalk in cancer progression and invasiveness. Among these, the bottom-up tissue engineering approaches envisage the assembling of building blocks such as cell sheets or engineered microtissues. The former are used in the so-called self-assembly methods of tissue engineering in which the self-production and assembly of cell-specific endogenous ECM components occur under ascorbate stimulation. A variety of tumor types, including those developing in the skin, bladder, and eye tissues have been developed with this approach [56]. On the other side, bioengineering approaches based on the use of engineered microtissues have been developed in our group, both by using single tumoral microtissue as in vitro cancer model [12,13], or by using engineered microtissues as building block in a modular tissue engineering approach [61]. The latter also takes advantage of endogenous cell-specific self-synthesized ECM and assembly, promoted by culture conditions in bioreactor, cell-materials interaction and ascorbate [65]. Recently, by following this approach we succeeded in developing an organotypic cervical cancer models by seeding organ-specific cancer epithelial cells on endogenous ECM synthesized, assembled and populated by normal or cancer-associated fibroblasts to investigate the role of diseased stromal environment in guiding the epithelial-mesenchymal transition process [61]. Taken together, these observations highlight the relevance of studying tumor cells in the correct physico-chemical context. The advantages of 3D cultures for developing disease models can be emphasized by coupling 3D models with OOC technology that allow precise and consistent cells positioning, integration with fluorescence confocal microscopy, microfluorimetry, transepithelial/transendothelial electrical resistance (TEER) measurements, multiple electrode configurations, and many other analytical tests [38].

Thanks to the technological progress in tissue engineering, [6,66] biomaterials, [67] and micro and biofabrication, [31,43,68], OOC aims at recapitulating the 3D organization and the multicellular complexity of tissues and enable enhanced dynamic control over the cellular microenvironment, leading to a new generation of biological systems with massive potential for drug screening and disease modeling.

### 3.2. Organ on Chip Technology Applied in Cancer: Cancer on Chip

**Figure 2:**
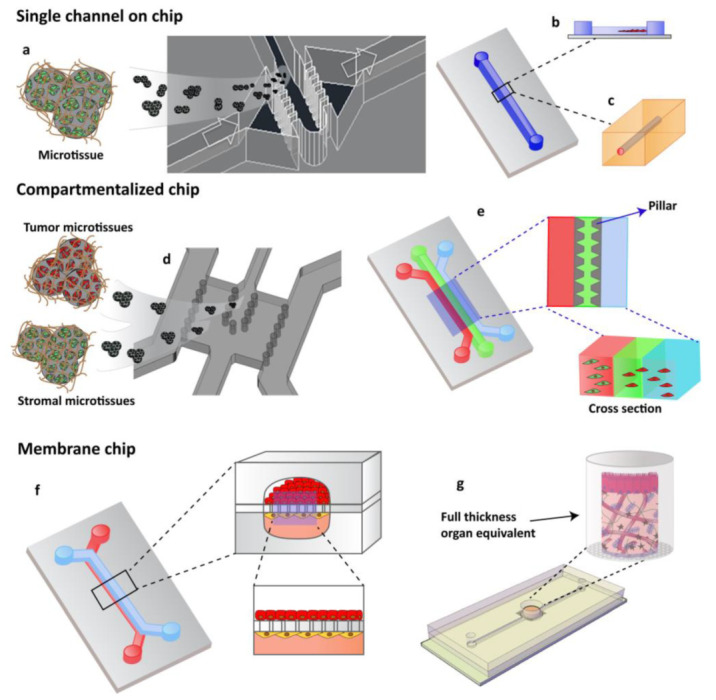
OOCs designs with different cell culture options. (**a**) Single channels chip designed by Garziano et al. to induce the assembly of dermal microtissues and on-line monitoring the newly synthesized collagen network, by means of SHG imaging, allowing to quantify in real time the effect of perfusion flow and biochemical stimulation on the newly collagen assembly degree. Reproduced from [96] with permission from The Royal Society of Chemistry. (**b**) Lumen channel designed to model human breast cancer cells collective invasion from primary tumors in response to interstitial fluid pressure, as reported by Piotrowski-Daspit et al. [87] or (**c**) designed to mimic a microvessel within a collagen gel scaffold as reported by Pauty et al. to study VEGF-A-induced angiogenesis permeability and angiogenic inhibitors effects [76]. (**d**,**e**) Compartmentalized chip: In these devices, pillars are used to separate microchannels in which 3D cell culturing is possible. The microchannels are independently addressable in order to fill each channel with a specific cell population and to allow heterotypic cultures. Depending upon the design of the chip, a 3D cell culture can occur by injecting the specific cell populated hydrogel in the correspondent channel or filling the microchamber with specific 3D microtissue. (**d**) The micropillar guarantees the physical contact of different cellular species embedded in their own ECM [93] or (**e**) in an ECM-like matrix. (**f**,**g**) Membrane chip. These devices allow a co-culture in a series of microchannels/chambers separated by a porous membrane. This multi-layered chip type was originally developed to mimic the endo- and epithelial cell layers found in the lung by Ingber [24]. Further, different devices have been designed in order to perform 3D organotypic culture by using both (**f**) membrane or (**g**) transwell insert.

OOCs have emerged in the last years as the new frontier in high-throughput screening technology, in drug assessment and development, and in other domains such as nutraceutics, and cosmeceutics identification. OOCs hold the potential to reduce animal testing and provide realistic human cell and tissue in vitro assays [69]. The objective of OOC technology is not to build a complete living organ, but to synthesize minimal units that recapitulate the functions at the level of tissues and organs [38]. In general, the OOC is provided with culture compartments, often separated by a porous membrane, in which 3D tissues, often consisting of several cell types, can be cultured while microchannels assure nutrient supply. OOC takes advantage of the recent development of microfabrication techniques, and can be “custom-designed” to better mimic tissue-specific function. Through the right choice and the design of materials in the chips and the introduction of electrodes to deliver electrical/mechanical stimuli, it is possible to recapitulate the tissue-specific microenvironment and control the behavior of cells. At the same time, the microchannels provide the cells with the necessary nutrients and remove waste and can be precisely engineered to assure the 3D tissues with the correct (bio)chemical environment, as in the body [57]. A sort of classification of OOC can be made by identifying: OOC designed as single channel, compartmentalized, or membrane chips-based system (Figure 2). The complete chips are typically a few cm in size and made up by optically accessible plastic, glass, or flexible polymers [57]. In addition to the choice of materials, stimulation, and sensing, the cell sourcing represents a key issue in OOC technology. Cells used in OOC come from three main sources: cell lines, primary cells from human donors, and human induced pluripotent stem cells (hiPSCs). To date, by using hiPSCs, primary cells, and cell line, several tissues and/or organ types have been successfully modeled to reproduce corresponding functional subunits, including, for example, the brain [30], heart [70], lung [10,25,71], liver [26,27], intestine [72,73,74], vasculature [75,76,77], kidney [78]. Importantly, these OOC devices can reproduce organ level response to exogenous agents such as inflammatory responses of the lung to silica nanoparticles, [25] intestinal epithelial-microbiome crosstalk, [73] early liver fibrotic activation in response to anti metabolites chemotherapy drug [79] as well as flow dependent recruitment of circulating immune cells [80,81], and organ specific inflammatory reaction in vitro. Moreover, they can also effectively mimic many types of organ specific disease states, including pulmonary oedema and thrombosis, asthma, inflammatory bowel disease, paving the way for a new era in drug development and new therapeutic discovery [16,31]. As oncology is one of the most important targets of drug discovery, its is in this area that a number of advances in the creation of more physiologically relevant approaches, such as COC, are most evident [16,57,82]. The typology of the cells used to produce COC is similar to that used in OOC. COC often uses cell lines but resulting in inconsistencies between the model and an original tumor. This limitation can be solved with the use of patient biopsies that would generate PDX-tumor on chip models that would represent more powerful models than current ones, avoiding the use of established lines. Regarding the use of iPSC, it has emerged as the most promising candidate for OOCs since it can be produced from almost every type of adult cell, including skin-, blood-, or hair cells, and can be used to produce many different cell types that are present in various organs of the body, and that would otherwise be very difficult to obtain, such as cells from the heart, brain, lung, liver, gut, and also blood vessels. Despite this extensive use in OOC, we still have very few iPSC-based COC. The two most crucial bottlenecks in the establishment of iPSC cancer models are the efficiency of malignant-cell reprogramming and the ability to differentiate iPSCs into the cell type of interest. A few published studies and anecdotal reports suggest that cancer cells are generally more refractory to reprogramming than normal cells [83]. Several COC models have been developed in the last years allowing one to manipulate the TME for studying cell behavior under specific metabolic gradients conditions [84] or to study the TME changes correlated to CAF interaction and vice versa [16,21,40,85,86]. Controlled parameters and read-out methods can be different among chip types, but the read-outs are commonly based on cell and invasive lesion tracking, [87] gradient sensing, staining, and gene expression quantification using RT-qPCR [39]. The COC community has devoted significant attention to visualizing ECM components and remodeling, for which, thank to the optical accessibility of the microfluidic devices, different microscopy and imaging techniques can be used, such as second harmonic generation (SHG), confocal reflectance microscopy and immunofluorescence [39,88,89,90]. This enables simultaneous interrogation of ECM composition and structure with the measurement of transport parameters. This is particularly advantageous when cell synthesized matrices are used in the microfluidic devices, since provide a more representative tumor ECM allowing for the quantification of the contribution of each ECM constituent to transport properties [88,91,92]. Some of the earliest applications of microfluidic cell culture technology focused on modeling specific steps in the cancer cascade, including tumor growth and expansion, angiogenesis, progression from early to late stage lesions involving an EMT, tumor cell invasion and metastasis. Here, we concentrate our attention on COC designed for recreating tissue–tissue interfaces, crucial for reconstituting in a physiological context the interaction occurring between tumor and its environment during cancer invasion and metastasis. Owing to several other interesting reviews looking into tumor chips including endothelial and immune cells [17,19,93,94,95] we do not discuss the tumor–endothelial–immune cells interactions in this paper but mainly debate cancer-cell–ECM interactions during tumor growth and invasion, focusing on the role of biophysical properties of ECM in guiding the pathological tumoral process.

#### 3.2.1. Compartmentalized Cancer on Chip for Modeling Tumor–Stroma Interaction

The complex microenvironment in which malignant tumor cells grow is crucial for cancer progression; therefore, modeling tissue-specific factors of the TME is crucial to creating physiologically and clinically relevant in vitro platforms for cancer research [19]. Multiple cell types can be cultured in a microfluidic chip and single-cell biophysical analyses of cancer cells has been implemented on chip, for detecting with high accuracy how specific physical properties of cancer cells (i.e., stiffness) vary with tumor progression and identify a malignant phenotype [97]. Moreover, it is particularly interesting and challenging culturing cancer cells and stromal cells in a microfluidic chip for investigating their communication during disease onset and progression [8].

**Figure 3:**
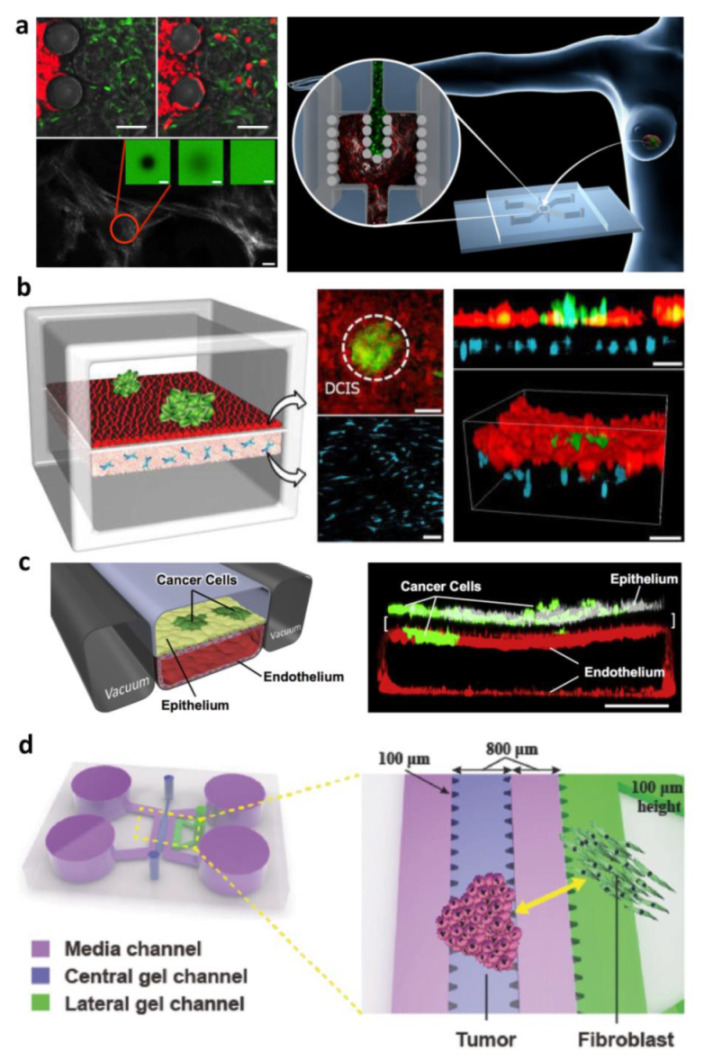
Cancer on chip for modeling tumor–stroma interaction: (**a**) On-chip activation of stromal tissue by crosstalk with cancerous tissue. The compartmentalized device is designed for accommodating stromal tissue and epithelial tumor tissue allowing microtissues physical contact, across the separation formed by the pillars, in order to replicate the tissue–tissue interface. SHG and FRAP techniques were used to investigate transport properties and remodeling of neo synthesized ECM and time-lapse images at fluorescence microscopy were used to detect the migration of MCF7 cells from the tumoral chamber to the stromal chamber. Reproduced with permission [92]. Copyright 2016, John Wiley and Sons. (**b**) Breast-cancer-on-a-membrane chip to replicate the early stages of breast cancer enabled the co-culture of multicellular ductal carcinoma in situ (DCIS) spheroids with normal epithelial cells close to human mammary fibroblasts embedded in a 3D ECM matrix. The DCIS spheroids were injected into the upper channel and adhered to the epithelial cell surface integrating into the epithelium. Reproduced from [14] with permission from The Royal Society of Chemistry. (**c**) Small airway membrane organ chips for orthotopically injecting a human non-small-cell lung cancer (NSCLC) line within the primary alveolus to recapitulate organ microenvironment-specific cancer behaviors. Reproduced with permission [10]. Copyright 2017, Elsevier. (**d**) Compartmentalized device with array of microposts enabling micropatterning of the cells-populated hydrogel. Cancer cells and normal fibroblasts were co-cultured in order to investigate both the role of fibroblasts in inducing morphological changes in cancer cells and if the hydrogel composition affects these changes. Reproduced with permission [103]. Copyright 2017, John Wiley and Sons.

In this perspective, compartmentalized COC models have been used to identify how neighboring normal parenchymal cells and ECM in the local tissue microenvironment can influence the progress of various types of cancer (Figure 3) [16].

##### Exogenous ECM-Based Compartmentalized COC

Injectable hydrogels, such as collagen I and Matrigel, are often used as 3D matrices to support cell growth and migration in microfluidic devices [39,98,99]. Several studies, aiming at investigating cancer-cell–ECM interactions in COC devices, compare Matrigel, collagen I, and a mixture of both to find the most appropriate matrix to study cancer invasion [100,101,102]. In this direction, by developing an Y chip, Sung et al. proved that non-invasive epithelial cancer cells that aggregate in 3D clusters and transform into an invasive phenotype need a blend made up by both gels [101]. Other relevant studies developed a compartmentalized microdevice to investigate the role of heterogeneous cancer cell subpopulations and of cancer fibroblasts in tumor progression and invasion correlated to ECM used. Shin et al. developed an in vitro breast tumor model to mimic intratumor heterogeneity in a microfluidic system with ECM scaffolds. They co-culture two breast cancer cell types with distinct phenotypes, specifically, highly invasive breast cancer cells with the high invasive potential and the capacity of proteolytic ECM remodeling (MDA-MB-231) and epithelial-like cancer cells (MCF-7) with a non-aggressive and low-invasive phenotype with strong cell–cell junction. The ability of MDA-MB-231 to promote MCF-7 invasion in the heterogeneous tumor mass was strongly dependent on the ECM type. They observed that MCF-7 cells only follow the invasion path of MDA-MB-231 cells when grown in Matrigel, but not when grown in collagen I [99]. In another work, Noo Li Jeon et al. examine cancer–stromal cell interaction with a 3D ECM, by using a microfluidic 3D cell culture platform in which an array of microposts enabled straightforward micropatterning of the hydrogel which allowed flexible experimental configurations [103]. They used ovarian adenocarcinoma (SK-OV-3), stomach cancer cells (MKN-74), and colorectal cancer (SW620) and normal human lung fibroblasts, and investigate both the role of co-culture with fibroblasts in inducing morphological changes in cancer cells and how the ECM composition affects these changes [103]. They found that fibroblasts induced marked morphological changes in all cancer cell types within 48 h in terms of increase in cytoplasmic volume and clustered nuclei. In addition, they observed that the co-culture effects of fibroblasts with cancer cells were greatly amplified in all cancer cell types under collagen–fibrin mixed ECM compared to fibrin alone indicating the synergistic effects of fibroblasts and ECM composition on cancer morphogenesis. The results highlighted the role of ECM composition, concentration, and stiffness in promoting cancer cells proliferation and in regulating their aggressiveness [103]. Other excellent examples of compartmentalized device showing how COC technology may be used to investigate with high resolution the complex interactions between multiple cancer-associated cell types and ECM molecules that are found in the local tissue microenvironment are proposed by Huh and co-workers to replicate the early stages of breast cancer. Their device enabled microfluidic co-culture of multicellular ductal carcinoma in situ (DCIS) spheroids with normal human mammary ductal epithelial cells in close apposition to human mammary fibroblasts embedded in a mixture of Matrigel and fibronectin. The DCIS spheroids were injected into the upper channel and successfully adhered to the epithelial cell surface and gradually became flattened and integrated into the epithelium. Prolonged culture in the microfluidic device resulted in enlargement of spheroids, indicating the ability of the model to support proliferation of DCIS cells. To emulate the physiological distribution of paclitaxel from the vascular network in the mammary stroma to ductal carcinoma, the authors established a dynamic fluid flow of a paclitaxel solution in the lower microchannel proving that under this condition, the growth of DCIS spheroids was inhibited compared with the significantly increased tumor volume without the drug. Moreover, they found that any toxic effects on normal mammary epithelium occurred [14,16]. In another study, by using a similar compartmentalized chip, Ingber’s research group demonstrated that by orthotopically injecting a human non-small-cell lung cancer (NSCLC) line within the primary alveolus and small airway organ chips, they were able to recapitulate organ microenvironment-specific cancer behaviors, including rapid growth in the lung alveolus microenvironment compared to relative tumor dormancy in the lung airway. They demonstrated that the rapid growth of NSCLC cells was correlated with some specific local microenvironmental factors produced by normal lung epithelial and endothelial cells put in contact across a porous ECM- coated synthetic membrane. Differently, the same cancer cells were not able to grow if cultured in 2D conditions with the same medium [10].

Other promising approaches to replicate the complexity of organ-specific ECM are based on the use of decellularized extracellular matrix. In this perspective, recently, Yi et al. created a patient-specific glioblastoma on a chip by printing cells encapsulated in a brain-derived decellularized extracellular matrix (BdECM) [104]. They created a COC provided with a compartmentalized cancer-stroma structure; to mimic the heterogeneous ecology of a glioblastoma, they build their model by surrounding the cancerous tissue with microvessels and induce the formation of central hypoxia by fabricating a device composed of selectively gas-permeable parts. They found that such a high degree of heterogeneity contributed significantly to the development of the various pathological features of glioblastoma-on-a-chip. Indeed, they observed that SOX2, a marker for neural stem cells implicated in the maintenance of cancer stem cells and therapeutic resistance of cancer cells, was observed only when endothelial and glioblastoma cells were grown compartmentalized and not when they are mixed. Moreover, they also observed that an ECM-like matrix used, collagen gel vs BdECM, affects glioblastoma cells behavior. Indeed, the cells cultured in BdECM proliferated faster, were more aggressive, had higher expression levels of ECM-remodeling proteins and proangiogenic factors, and showed increased formation of vascular networks. Further, glioblastoma cells in BdECM showed also different drug sensitivities, compared to cells within collagen. Finally, with transcriptomic profiling and bioinformatics, Yi et al. showed that the glioblastoma-on-a-chip could be used to find drug sensitivities and create an effective patient-specific therapeutic approach in a clinically relevant timeframe. Through the integration of patient-specific cancer cells, organ-specific native ECM, cell compartmentalization, the glioblastoma-on-a- chip closely emulates the pathological features of glioblastoma and has the potential to hold the promise of developing a more precise cancer medicine [104].

##### Endogenous ECM Compartmentalized COC

Results obtained with hydrogel-based cancer models show that the 3D microenvironment and its biochemical properties are crucial for replicating the interactions between cells and the ECM. Indeed, the type of ECM used in 3D cell culture matters and the conventional hydrogels, often, barely mimic the complex molecular composition of the native ECM and its associated interactions with cells. Reproducing specific human matrix microenvironment in vitro, including the proper ECM composition and organization, is a challenge and developing process aiming at inducing the cells to produce their own ECM, have emerged as a promising alternative. In this perspective, our group developed healthy and tumor models in which cells are embedded in their own ECM. We integrated our tissue models in COC to replicate the interactions of breast cancer cells with stromal cells as well as ECM activation during tumor progression [92]. We designed an optically accessible microfluidic chip with two compartments for hosting stromal tissue and epithelial tumor tissue, respectively, separated by an interface that allowed their physical contact in order to replicate the tissue–tissue interface. In contrast to the aforementioned works in which cells are embedded in 3D exogenous matrix (collagen, fibrin, or mixtures of both), in our model, the 3D stromal tissue consisted of engineered tissue micromodules formed by fibroblast-assembled ECM. We have been previously demonstrated that such engineered tissue micromodules replicate the tumor physiology in vitro including functional and morphological changes [12,64,91]. Then, in our compartmentalized microdevice, we proved that the stroma tissue underwent both cellular and extracellular activation during breast cancer cell invasion. Dormant fibroblasts differentiate to activated fibroblasts while ECM showed fibronectin and hyaluronic acid overexpression. In addition, analysis of collagen texture showed a change from the fine fibril structure, which featured healthy stromal tissue, toward a coarser network, that is, a finding consistent with reports for human biopsies of epithelial tumors. At last, we modeled drug delivery to a tumor by diffusion by using Fluorescein isothiocyanate (FITC)-dextran, finding a significantly lower diffusion coefficient for the activated stroma, compared to the healthy one. We hypothesized this behavior was correlated with the increased frictional interaction between dextran and the ECM because of coarser collagen fibers, suggesting that anticancer drugs relying on diffusion to reach cancer tissue might have poor penetration and limited therapeutic efficacy. Our results demonstrated that such COC allowed to capture ECM dynamics and model drug delivery supporting scientists to evaluating the efficacy of such a treatment approach [2].

#### 3.2.2. Multi-Organs System for Modeling Cancer Metastasis and Toxicity

Organ and cancer on chip models are very promising in vitro platforms to unravel unknown patho-physiological in vivo behavior and finding novel therapeutic approaches. However, human tissues and organs do not exist in isolation in the body, but are in constant communication. This cross-communication is essential for providing tissues with correct signals and support from other districts and guarantying normal tissue viability and function. With respect to a new drug, for example, its real therapeutic value or its side effects cannot be estimated effectively unless it has been tested in a system more complex than just one organ [6,16,42,105,106,107,108]. Similarly, in cancer metastasis multiple tissue or organ sites as well the circulatory system is engaged (Figure 1). As such, although useful for many applications, single COC models cannot recapitulate efficiently the many types of interaction between multiple tissues occurring in the human body [6].

**Figure 4:**
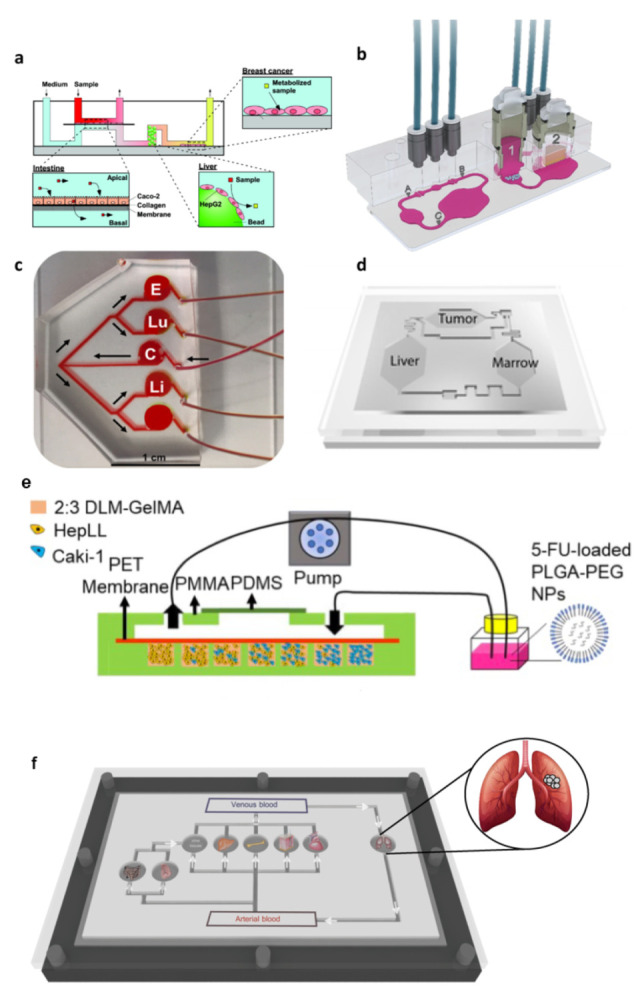
Multiorgan on chip for modeling cancer metastasis. (**a**) An intestine-liver-tumor multiorgan device provided with systemic and intestinal perfusion. Reproduced with permission [106]. Copyright 2010, American Chemical Society. (**b**) Hybrid Multi-Organ-Chip to replicate human tumor–skin co-culture assay to evaluate anti-EGFR antibody effects on lung tumor spheroids and human skin. The chip’s optical accessibility allows the tracking of the metastatic outgrowth of the tumor microtissues through daily imaging and facilitates in-depth fluid flow analyses at spots A, B and C on the chip. Reproduced with permission [107]. Copyright 2018, Springer Nature. (**c**) Metastasis-on-a-chip device. Media is perfused into the device at the single inlet port into the colorectal cancer chamber (C) from the media reservoir from the micro peristaltic pump. From the cancer chamber, the channels bifurcate twice, providing equal flow to the endothelial (E), lung (Lu), liver (Li) constructs. Reproduced with permission [110]. Copyright 2019, John Wiley and Sons. (**d**) The Micro cell culture analog device with 3-D hydrogel cell cultures has been developed to test the cytotoxicity of anticancer drugs while reproducing multi-organ interactions. It accommodated liver, tumor, and marrow chambers, interconnected with channels mimicking the blood flow pattern in the human body. Reproduced with permission [109]. Copyright 2008, The Royal Society of Chemistry. (**e**) Simulation of the biomimetic liver microenvironment in a tumor progression model based on metastasis-on-a-chip. Caki-1 cells have been cultured in 3D biomimetic liver microenvironments (2:3 DLM/GelMA) to mimic the progression of metastatic kidney cancer. The efficacy of 5-FU, delivery of 5-FU though PLGA-PEG NPs, and dose optimization have been measured [111]. (**f**) A vision of body on chip in which different organs can be cultured. In vitro three-dimensional models in cancer research: a review, Imparato G, Urciuolo F, Netti PA, International Materials Reviews, 2015, Taylor & Francis Ltd., reprinted by permission of [6] Taylor & Francis Ltd.

Thus, microengineered tumor culture could integrate with multi organ microdevice technologies for comprehensive culture models of disease progression across multiple tissues (Figure 4). These microfluidic devices are designed to mimic important aspects of the human metabolism by interconnecting fluid flows from multiple in vitro tissue cultures on the chip in a physiologically relevant manner so that metabolites are consumed, produced, and exchanged (via recirculation) between all tissues at physiologically relevant concentrations. One of the first experiment to show how multiple-organ systems can mimic human responses to drugs were performed by Sung et al. with their microphysiological system containing 3 types of organoids—liver, bone marrow, and a tumor of the colon—on a single chip with closed circulation. They investigated the metabolism of Tegafur a prodrug of 5-fluorouracil (5-FU), an anticancer drug. Tegafur is a molecule more stable than 5-FU; when Tegafur is metabolized by the body it becomes the active drug. By itself, Tegafur was not toxic to the patient or the cancer; but when metabolized by an enzyme in the liver, it became effective against the cancer and remains active in the body much longer than oral 5-FU did. By using the multi organ microphysiological device, Sung et al. reproduce the way the liver metabolizes the Tegafur, and demonstrated that the Tegafur itself did not harm the colon cancer organoid, but after passing in the liver, the resulting active drug resulting fatal for cancer cells [109]. Further, novel multiorgan on chip platforms have been developed to investigate how drugs work inside the body, find new therapies, and better understand cancer metastasis [37]. In metastasis, after undergoing EMT transition, cells proliferate very fast in the primary tumor site and then intravasate through endothelium into the vascular or lymphatic system, after which they extravasate and migrate toward a secondary tissue site, usually located downstream from the primary site. It appears evident the importance that a multi-organoid approach can have to model the kinetics of metastasis in vitro. Currently, several metastasis-on-a-chip platforms are in development, and some researcher groups have demonstrated the possibility to recapitulate metastasis in vitro, even if in a reductionist manner. One such microfluidic platform was created to ease the tracking of the migration of the metastatic tumor cells from a colon organoid to a liver organoid under recirculating fluid flow [41,110]. The results confirmed in an in vitro model the ability of metastatic colorectal cancer cells (CRC) to migrate and disseminate out of the colon organoid into the circulating perfusion system and colonized the down-stream liver organoid. In contrast, any migration, but only proliferation, occurred when non-metastatic colorectal cancer cells were used [41]. Recently, the same group implemented the platform to accommodate also lung and endothelial tissue constructs, demonstrating the preferential attitude of CRC cells of homing to the liver and lung constructs, in agreement with the clinical situations in human patients [110].

Another example of metastasis-on-a-chip incorporating with organ-specific ECM have been developed by Wang et al., with the aim of mimicking the progression of kidney cancer cells in the liver to predict the therapeutic effects and evaluate dosage responses of anticancer drugs [111]. They cultured kidney cancer cells (Caki-1) and hepatocytes (i.e., HepLL cells) in a decellularized liver matrix (DLM)/gelatin methacryloyl (GelMA)-based liver microtissue in the metastasis-on-a-chip device. By using HepLL cells in increasing ratios to investigate the metastasis progression process of kidney cancer cells in the liver, they observed that there was a linear anticancer relationship between the concentration of 5-FU and the number of Caki-1 cells, and that the 5-FU-loaded PLGA-PEG nanoparticles showed a higher capability of killing tumor cells than free 5-FU. The established 3D metastatic cancer in vitro models could be used to rapidly assess anti-cancer efficiency and optimize dosage regimes [111]. Another notable example of multi-organs-on-a-chip to elucidate the mechanism underlying organ-specific cancer metastasis was developed by Xu et al. to recapitulate the tissue−tissue interfaces and complex function of lung and distant organs. Indeed, lung cancer usually metastasizes to the bone, brain, and liver, contributing to a bare prognosis. The existing lung on chip provides lung cell with a physiological culture microenvironment but are not able to reproduce critical steps such as transition, invasion, and metastatic progression of lung cancer. Xu et al. developed a multi-organs-on-a-chip to investigate lung cancer metastasis to the brain, bone, and liver, and to analyze the cell physiology and cell−cell interactions in a more physiologically relevant context. They cultured bronchial epithelial, lung cancer, microvascular endothelial, mononuclear, and fibroblast cells divided by membrane in the “lung” chamber, while astrocytes, osteocytes, and hepatocytes were cultured in distant chambers, emulating the metastatic process of lung cancer cells in the brain, bone, and liver, respectively. They found that lung cancer cells formed a “tumor mass” after culturing in this system, forming an epithelial-mesenchymal transition (with modified expression of E-cadherin, N-cadherin, Snail1, and Snail2) and exhibited a high invasive capacity [112].

However, the majority of multi-site metastasis-on-a-chip platforms included two organs, representative for primary and secondary tumor sites, respectively. They are designed to assess specific aspects of metastasis, enabling, for example, the control over parameters affecting tumor cell migration and real-time monitoring of the cancer invasion process. Since myriad parameters are involved in cancer metastasis, providing an in vitro model representing the metastasis process, even in its reduction form, can bring new insights to understand, predict, and control this cancer progression mechanism [113].

In this perspective, a very simple device, was developed by Hao et al. for bone metastasis study of breast cancer cells. In this bone-on-chip microfluidic device, a spontaneously formation of mature mineralized osteoblastic tissue occurred in 30 days, by optimizing the features of the cell culture chamber (i.e., pore membrane diameter, height of chamber) and by assuring access to fresh nutrients, the prompt removal of metabolic waste and the high local concentration of bone matrix building protein. The authors highlighted that the resulting physiological relevant natural bone microenvironment composed of cell-synthesized, mineralized ECM cannot be achieved by seeding cells within synthetic exogenous scaffold. By co-culturing metastatic breast cancer cells with the osteoblastic tissue inside the bone on chip, they found unique hallmarks of breast cancer bone colonization previous observed only in vivo, demonstrating the relevance of replicating a native endogenous TME, without synthetic scaffold, to mimic the metastatic process [114].

In another work, a breast cancer-to-bone metastasis on a chip was developed. An endothelial cell layer that acts as a vascular barrier to a chamber that mimics 3D bone, allowing researchers to model extravasation of circulating breast cancer cells into bone [115]. The results show that host chemokines had a great impact on attracting tumor cells toward the bone microtissue [10].

In a more recent work, an hepatocellular carcinoma (HCC)–bone metastasis on chip was developed to model and track the metastatic cells and to analyze the inhibitory effect of an herb-based compound, thymoquinone (TQ) (in free form or encapsulated in chitosan nanoparticle), in hindering the migration of liver cancer cells into the bone compartment. Biomimetic 3D hydrogel loaded with HepG2 emulated the primary hepatic tumor tissue in one compartment while a bone-mimetic niche, composed by a 3D hydrogel matrix containing the bone mineral hydroxyapatite (HAp), was created in the secondary tumor site compartment. A microporous membrane was placed above the compartments to resemble the vascular barrier, and the medium was circulated over the membrane. It was observed that the liver cancer cells proliferated and disseminated from the HCC chamber to the circulatory flow and eventually entered the bone chamber. When Hap was present in the hydrogel, the number of metastatic HepG2 cells to the bone compartment increased, suggesting that the calcium ions released from the chamber containing HAp affects the HepG2 transmigration and settling in the secondary bone mimetic site. This behavior highlights the relevance of a specific TME in guiding the metastasis process in a HAp-dependent manner. Moreover, the results on the metastasis-controlling effect of TQ show that TQ-encapsulated nanoparticles could inhibit HCC metastasis for longer duration in comparison with the case in which free molecules were administrated. Taken together, the results demonstrated that the HCC–bone metastasis-on-a-chip platform can model certain key aspects of the cancer metastasis process, hence corroborating the potential of enabling investigations on metastasis-associated biology as well as improved anti-metastatic drug screening. Several commercial microfluidic devices are nowadays available; among these, a multi-organ chip platform linking two organ culture compartments was adapted by Hübner et al. for microfluidic co-culture of human H292 lung cancer microtissue and human full-thickness skin equivalents to evaluate the effect of anti-EGFR antibodies (cetuximab) on both tumor and human skin tissue. The latter is the site of target-mediated adverse effects in patients. They found that repeated dose treatment of the cetuximab r increased the pro-apoptotic related gene expression in the tumor lung microtissues. At the same time, proliferative keratinocytes in the innermost layer of the epidermis of the skin equivalent were eliminated, revealing crucial inhibitory effects on the physiological epidermal cell turnover. The combination of a metastatic tumor environment with a healthy organotypic human skin equivalent make the multi-organ device an ideal tool for the simultaneous generation of safety and efficacy data [107]. In another work, a vascularized breast tumor and healthy or tumorigenic liver microenvironments were connected in series on-chip to allow for the study of dynamic and spatial transport of particles. The device enabled the dynamic determination of vessel permeability, the measurement of drug and nanoparticle transport, and the assessment of the associated efficacy and toxicity to the liver [116]. The reported examples demonstrate an important contribution of OOC technology in creating tissue-specific experimental models of the metastatic niche that are strongly needed to identify the critical factors correlated with the metastatic cell homing and colonization at distant sites, as well as the tumor resistance to treatment.

## 4. Coupling Cancer on Chip and Artificial Intelligence for Future Cancer Management

The examples reported above highlight that the COC technology enables modeling of various types of cancer, studying cancer pathology, progress, and response to various therapeutic agents. COC are characterized by the ability to recapitulate complex cellular and extracellular microenvironment of tumors and allowed to investigate the role of various microenvironmental features occurring during different stages of cancer metastasis.

In this perspective, the harnessing of COC models and AI seems have a great potential in delineating the TME by identifying novel features and analyzing and interpretating multiomics data in a objective, reproducible, and efficient manner, overcoming the limitation of previous methods of TME analysis.

Currently, automated systems are utilized in a clinical setting to identify specific target cell types, and the digitization of histopathological images has enabled more precise analyses. The resulting data can be input into machine learning (ML) algorithms for pattern recognition without the need for frequent fine-tuning of parameters or supervision [117]. Moreover, the development of high-throughput whole-slide imaging technologies has opened avenues for the ML analysis of immunohistochemical (IHC) source data to yield large datasets for precision oncology [118]. Thus, compared with the more traditional approach to IHC pattern analysis at the cellular level, ML models enable a more holistic analysis of the TME at the whole-slide level [119]. AI is already being used to analyze various tumor types, such as breast and lung cancers [120], and is poised to elevate histological analysis to a more sophisticated level. Given the established strengths of ML techniques in IHC, the potential application of AI in the analysis of tumor models and COC technology is an exciting prospect. Studies using COC have yielded huge quantities of data, and Elmusrati and Ashammakhi [36], as well as Fetah et al. [33], have proposed the use of ML models to extract and maximize this information. Indeed, the use of COC technology allows for the integration of complex assays and non-invasive real-time monitoring of important cellular and extracellular parameters exploiting for example advanced imaging technologies. Therefore, they represent high-throughput systems that will help to derive sound conclusions on the basis of a massive amount of data. The latter need for appropriate data management and analysis system. Therefore, in parallel to the production of novel COC platforms, ML algorithms to manage these data have been developed. In addition, although traditional ML offers advanced data processing capabilities, the advent of its most important component, the deep learning, made possible to analyze massive unstructured data such as images, drug–target interactions, and computational biology [16,17,18,19,34]. An example of combination of advanced live cell imaging algorithm and artificial intelligence with COC technology is reported by Oliver et al. [35] They have developed a platform for detecting cancer cells with a brain metastatic phenotype combining AI, a blood–brain barrier on a chip and confocal tomography to discern between the metastatic signatures of cancer cells. Using their chip, they typify the migratory and proliferative phenotypes of cancer cells having varying degrees of brain metastatic potential as well as cells from cancer patient samples with known metastatic potential. By combining these results with AI, it is possible to predict the metastatic potential of cancer cells. Recent evidences report that diagnosis based on SHG images and ML can support the rapid and accurate detection of some kinds of cancer in clinical practice [121]. COC device hosting engineered tumoral tissue presenting native ECM (cell-synthesized or cell-derived) have been already used to be analyzed by SHG-MPM to detect pathological alterations to the ECM. Therefore, they are a promising platform to implement ML into the SHG image postprocessing to enhance differentiation of normal and tumor tissues, potentially enabling automated screening of tissue in COC under a different therapeutic regime. Nonetheless, the integration of ML algorithms for microfluidic tumor model analysis remains in its infancy, with few demonstrated examples. Several limitations of AI in the analysis of tumor models have to be overcome, including the lack of sufficient, high-quality datasets. Further research is thus warranted, to enable us to harness the full potential of bringing together in vitro models and AI in the study of the TME and to enable us to extract and maximize the vast quantity of information stored within the intricacies of the TME. This translates into improved diagnostic, prognostic, and therapeutic outcomes for patients [122].

## 5. Pros and Cons of Cancer on Chip

There is a wide range of tumor models, each with distinct advantages and disadvantages. Due to the inherent differences in complexity and functionality, the choice of model is usually dependent on the application. For example, compartmentalized COCs replicate the pato-physiological separation existing between cancer mass and stroma allowing to perform advanced invasion assays to see how cancer cells cross the barrier and interact with the stromal population. [59] In this perspective, compartmentalized COCs, faithfully recapitulate the TME and cell heterogeneity and allow one to simulate the endothelial network, resulting as being particularly useful in studies focusing on the neovascularization, invasion, and dissemination of cancer cells [38]. In general, COCs models are less costly than animals; pre-clinical models do not present ethical concerns and solve the differences that exist between humans and animal species that make the translation of preclinical results from animals to humans not always possible. Despite standard 3D cell culture, COCs present the advantage not only to better recapitulate native human microenvironments, but also to be provided with electrodes and sensors allowing one to model and control mechanical stresses, fluid flow, oxygen levels, temperature, and pH [97]. At the same time, however, COCs are more difficult to use than many other 3D culture systems and their use needs highly specialized personnel which can result in increasing experimental costs. However, compared with standard 3D human macroscale models, the addition of the chip allows the obtaining of high-resolution images that allow one to determine where in the tissue to look. Technical robustness is another challenge, as the small scale and complexity of microfluidic systems that experience controlled fluid flow require that many factors must interplay perfectly to achieve optimal functionality, and simple factors, such as bubble formation, can ruin an experiment. For long-term studies, there is the challenge of maintaining cell viability and functionality and structural integrity of multiple tissues and different cell types using a common media and consistent fluid flow [123,124]. In addition, the use of COCs do not allow one to recreate tumor at real size (typically > 10^9^ cells) but it is possible to reach a cellular range of 10^6^ cells and more importantly, to recapitulate cellular heterogeneity which is a fundamental feature of tumor behavior. At last, it is to be noted that, until now, while extraordinary advances have been attained in integrating a miniaturized tool for controlling and sensing directly on-chip, the design and realization of tissue engineered constructs generally used on the chip is still very poor. The generalized use of exogenous materials as cell scaffolding along with a poor control of microenvironmental condition at single cell level, strongly limit COC current reliability and robustness. The next development in this field aims at designing COC devices able to take into account not only the three-dimensionality of the tissue, but also the time and space molecular presentation and morphophysical features of the cell microenvironment in tissue (patho-) physiology. This is strongly required, particularly, to mimic events in which ECM dynamics play a pivotal role such as the development process, aging, fibrosis, desmoplastic reaction [125].

## 6. Concluding Remarks

The integration of multiple tissue components is needed to recreate the complex TME in which the tumorigenesis starts and evolves. To mimic such a complex process, reductionist 2D or 3D models, lacking tissue structure and tissue/multicellular pato-physiological crosstalking, cannot be used. OOCs represent physiological, humanized models that allows personalization to specific cancer types and integration of multiple systems. They also allow real-time and non-invasive monitoring of cell-based assays with tissue and organ level complexity.

Their implementation in various areas of basic and translational cancer research provides a unique opportunity to analyze and mimic remarkably complex physio-logical processes. The integration of tumors into OOC platforms will enable robust and predictive in vitro modeling of human pathophysiology. Recently, patient-derived multi-cellular spheroid system have been recognized to be very promising in personalized medicine since they can be obtained from patient tissue and can be used in high-throughput personalized medicine methods, providing a suitable therapy for that patient [126].

Similarly, the use of patient biopsies could be implemented to generate small-scale PDX tumor-on-a-chip models representing more powerful models than current ones and avoid the use of established cell lines. However, the design of a realistic model seems to focus not only on cell composition and origin, but also increasingly, on the general microenvironment and the ECM that composes it. New approaches aimed at emphasizing the importance of the TME and cell–cell crosstalk in cancer progression and invasiveness are based on obtaining the ECM from decellularized human biopsies or combining cancer and cells in the self-secreted stroma (both from healthy and tumor tissue), rather than using materials from another natural or synthetic source [35]. The shift from OOC to body on chip is only a matter of time and the implementation of increasingly complex vascular networks and target organs will owe much to the development of new and more powerful tools. It will take open-mindedness from researchers, funders, and regulators to encourage the adoption of OOC as an alternative to animal models for in vitro testing. The recent commercialization of these technologies by several companies should allow many academic, industrial, government, and clinical groups to explore the value of this approach in their own laboratories. Therefore, the coming years will bring a paradigm change in drug development, disease modeling, and precision medicine as tumor chip models are widely used in academia, industry, and healthcare.
